# Natural sex reversal imparts permanent compositional changes to the swamp eel gonadal microbiome

**DOI:** 10.1186/s40168-025-02187-7

**Published:** 2025-10-24

**Authors:** Kaifeng Meng, Meidi Hu, Yuanyuan Chen, Xing Lin, Chaolin Jiang, Jiarui Song, Yifan Bai, Yuanli Zhao, Fei Liu, Daji Luo

**Affiliations:** 1 State Key Laboratory of Breeding Biotechnology and Sustainable Aquaculture, Institute of Hydrobiology, Hubei Hongshan Laboratory, Chinese Academy of Sciences, Wuhan, 430072 China; 2 College of Fisheries, Huazhong Agricultural University, Wuhan, 430070 China; 3 Fisheries College, Ocean University of China, Qingdao, 266001 China; 4 College of Advanced Agricultural Sciences, University of Chinese Academy of Sciences, Beijing, 100049 China

**Keywords:** Microbial community, Gonad, Hermaphroditic fish, *Bacillus*, Sex differentiation

## Abstract

**Background:**

Microbial communities are increasingly recognized for their essential roles in the reproductive system. However, the microbial communities in healthy gonads—neither in the ovary nor the testis—have not been extensively explored, particularly with respect to sex differentiation. Sex reversal is a unique mode of sex differentiation that is a well-documented phenomenon in various animal species, with the swamp eel (*Monopterus albus*) being a notable example of a hermaphroditic species that undergoes natural female-to-male sex reversal. Thus, swamp eel offers a robust system for exploring gonad microbial communities and their biological and functional significance.

**Results:**

Our study revealed a living microbial community in the gonads of healthy swamp eel, with microbial loads comparable to those found in three distinct niches: gut, skin, and blood. The gonad microbial communities shared > 55% of their diversity with those in the gut and blood. We focused on the niche-specific differences in microbial communities, particularly between the ovary and testis. After isolating and injecting the ovarian-dominant bacteria *Bacillus*, we observed significant microbial dysbiosis and metabolic responses in the ovary. These changes were primarily reflected in the altered abundance of the ovarian microbiota involved in amino acid and lipid metabolism, which may contribute to ovarian function in swamp eel. Additionally, *Bacillus* inhibited sperm motility, reduced sperm count, and induced inflammatory responses in the testes of male swamp eel. These findings highlight the crucial role of bacteria in the sexual transition from the ovary to the testis and in gametogenesis.

**Conclusions:**

Characterizing the microbial composition and distribution in the gonads is crucial for understanding the role of the reproductive microbiome in hermaphroditic species and during sex reversal. Our findings first indicate that ovarian-dominant bacterial communities contribute to maintaining ovarian function while inhibiting testicular function in swamp eel, further suggesting that microbial communities are involved in the process of sex reversal.

Video Abstract

**Graphical Abstract:**

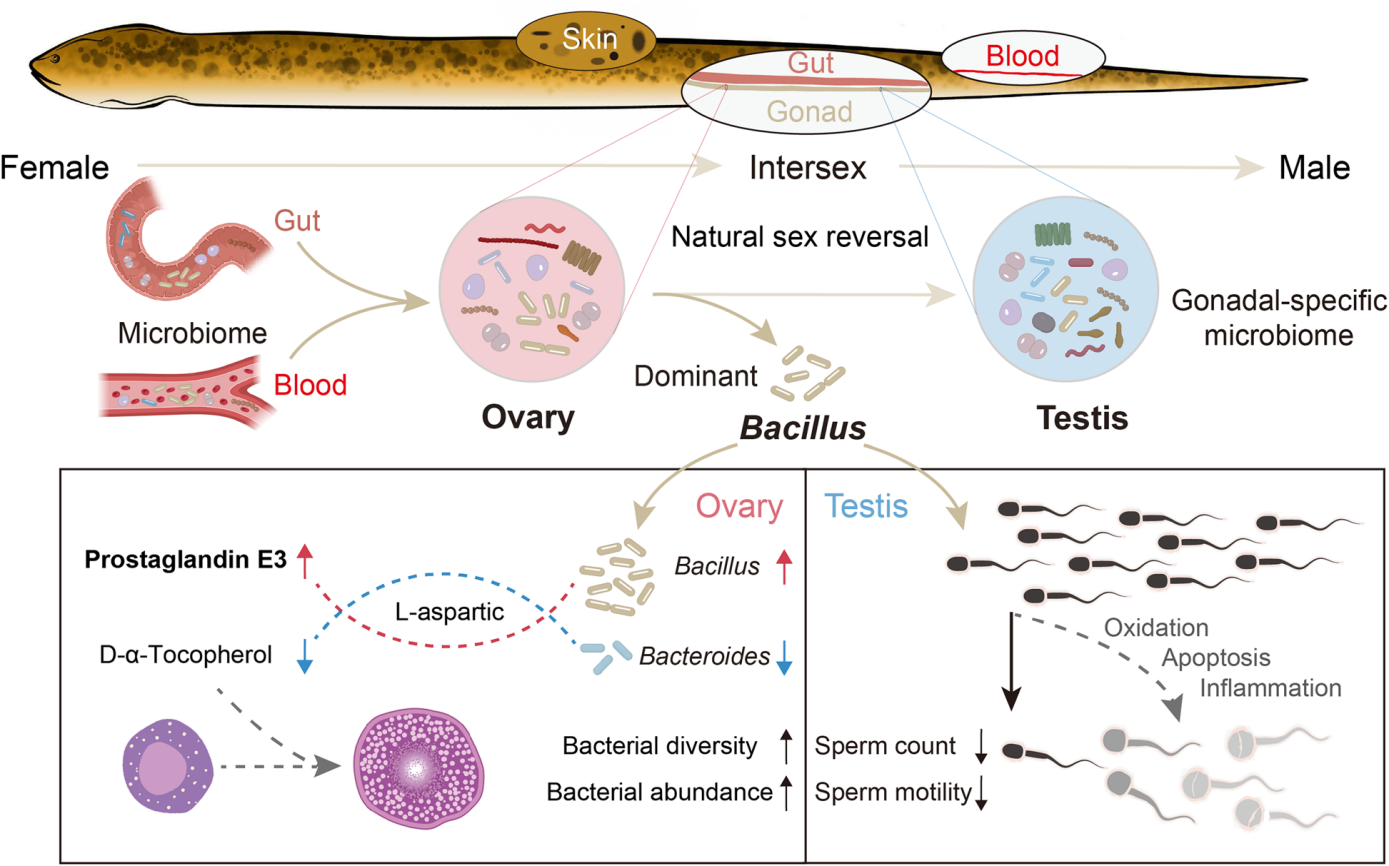

**Supplementary Information:**

The online version contains supplementary material available at 10.1186/s40168-025-02187-7.

## Highlights

1. Gonadal microbial communities at homeostasis are partially derived from the gut and blood microbiomes.

2. The dominant ovarian bacteria *Bacillus* leads to ovarian dysbiosis.

3. Prostaglandin E3 may serve as a metabolic biomarker in response to *Bacillus*.

4. *Bacillus* induces testicular inflammation and reduces sperm motility in hermaphroditic swamp eel.

**Supplementary Information:**

The online version contains supplementary material available at 10.1186/s40168-025-02187-7.

## Background

Distinct microbial communities have been identified in both sexes at multiple locations along the reproductive tract, exhibiting variations in bacterial diversity and composition [1, 2]. Microbiomes are increasingly acknowledged for their significant roles in the reproductive system [3]. Although some microorganisms are directly transferred from the reproductive tract of the mother to the baby during birth [4, 5], the microbial communities in healthy gonads—neither the ovary nor the testis—have not been extensively characterized, particularly in relation to sex differentiation and gonadal development. Teleosts, in particular, seem to be especially receptive to the presence of bacteria in their internal organs [6, 7]. While evidence is accumulating for the presence of microorganisms in the gonad [8], the question of whether gonadal microbiomes maintain homeostasis remains unanswered.

Sex reversal, an extraordinary form of sexual plasticity that occurs during the life cycle, has been studied in fish, reptiles, birds, amphibians, and even mammals [9]. The swamp eel (*Monopterus albus*), a typical protogynous hermaphrodite fish, undergoes natural sex reversal, where an ovary transforms into a testis through ovotestis differentiation during its life cycle [6, 10]. While reproductive tract microbiomes are well characterized in female vertebrates, particularly the vaginal and cervical microbiota dominated by *Lactobacillus* species, emerging evidence suggests that even low-biomass ovarian microbial communities may influence reproductive health [11, 12]. In addition to the female system, gut-resident microbes such as *Bacillus* enhance reproductive performance through immunomodulation and metabolic regulation, improving folliculogenesis and fetal development [13]. Similarly, the seminal microbiota critically regulates physiological and pathological processes in male models [14, 15]. However, the presence of microbial communities in the vertebrate testis remains highly controversial, with reported associations limited to disease states [16], heat stress [17], and teleost-specific adaptations [6, 18]. Microbial communication via metabolites and signaling molecules is known to maintain tissue homeostasis [19]; however, its role in orchestrating gonad plasticity remains entirely unexplored. We hypothesize that dynamic microbiota-gonad communication facilitates sex reversal in teleosts, with microbial-derived cues potentially acting as modulators of this extraordinary sex differentiation.

To investigate microbiota-gonad communications in hermaphroditic swamp eel, we integrated multi-omics analyses to resolve functional interactions between the symbiotic microbiota and gonadal development. Here, we demonstrated that sexually dimorphic bacterial communities colonize healthy ovaries and testes, with partial transmission from the gut and blood microbiota. Strikingly, functional analyses revealed that *Bacillus* supplementation dynamically reshaped ovarian microbial networks, upregulating taxa enriched in amino acid and lipid metabolism pathways critical for follicular maturation. Conversely, in males, *Bacillus* triggered testicular inflammation and impaired spermatogenesis, which was characterized by reduced sperm motility and dysregulated immune signaling. These findings highlight a dual role for the microbiota in modulating gonad plasticity: while the ovarian microbiota may prime metabolic pathways essential for female function, testicular communities appear sensitive to microbial-driven inflammatory responses. Notably, our findings provide the first evidence that the microbiota directly influences gonad homeostasis in a sex-reversal vertebrate. By connecting microbial metabolites to gonadal remodeling, this work establishes a framework for understanding how microbiota-encoded signals could orchestrate gonadal functions during natural sex reversal. This finding opens up new avenues for exploring microbiota-driven modulation of gonadal homeostasis.

## Results

### Viable bacteria were present in both the ovary and the testis of laboratory-reared swamp eel

**Fig. 1:**
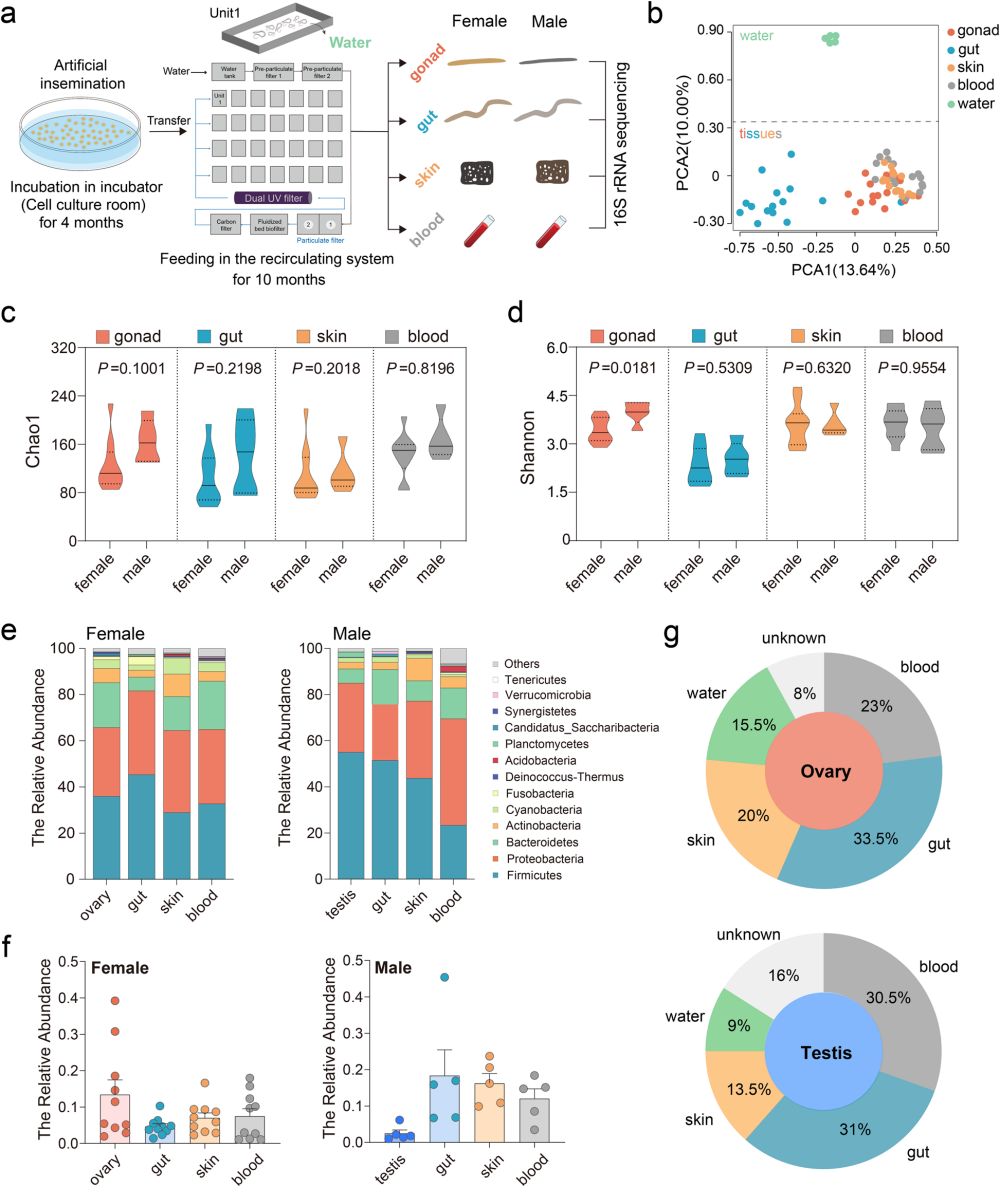
Distinct bacterial characteristics in different niches between female and male swamp eel. **a** Experimental design overview. **b** PCA showing the microbiota community from water and swamp eel. **c** Richness of the bacterial community in female and male swamp eel. **d** Diversity of the bacterial community in female and male swamp eel. **e** Stacking diagram showing the composition analysis of the bacterial community at the phylum level in female (left) and male (right) swamp eel. **f** Predicted relative percentages of the bacterial community in the ovary and testis originating from the blood, gut, water, skin, or unknown sources via Sourcetracker2 analysis. **g** Representative abundance of *Bacillales* at the order level in female (left) and male (right) swamp eel. Statistical differences were evaluated by unpaired Student’s *t* test. *p* values < 0.05 or less were considered statistically significant

To investigate the interrelationships between the bacterial community and gonadal development in swamp eel, we obtained the F1 generation through artificial insemination to eliminate the interference from the genetic background. After being maintained in the indoor recirculating system for 10 months, four tissues, including gonad (ovary and testis), gut, skin, and blood were collected, and the abundances of their microbiota communities were employed via 16S rRNA sequencing (Fig. 1a). After quality filtering and normalization to remove adaptors and low-quality or ambiguous bases, a total of 1747 operational taxonomic units (OTUs) were identified in the four groups. Subsequently, 251 common OTUs and 224, 164, 184, and 345 specific OTUs were detected in the gonad, gut, skin, and blood, respectively (Fig. S1a). Principal component analysis (PCA) was performed to determine the relationships among the samples. A highly significant separation between samples of different groups and water was observed. Moreover, the samples from the gonad, skin, and blood overlapped with little separation. They were significantly separated from the gut (Fig. 1b; Fig. S1b). According to the microbiota alpha diversity analysis calculated by the Chao1 and Shannon diversity indices, there was no significant difference among the gonad, gut, skin, and blood samples. However, the Shannon index in the testis was significantly (*p* = 0.0181) greater than that in the ovary, whereas the Chao1 index was slightly (*p* = 0.1001) greater than that in the female group when sex was considered (Fig. 1c, d; Fig. S1c), suggesting that the microbial environment may be unique between the testes and ovaries.

The obtained bacterial communities were further divided based on sex. The results revealed that thirteen phyla were predominant among all samples (Fig. 1e). Firmicutes was the most abundant phylum in both the female and male groups, with tissue-specific distributions as follows: gonads (female 35.9%, male 55.0%), gut (45.2% vs. 51.4%), skin (28.8% vs. 43.7%), and blood (32.6% vs. 23.2%). Proteobacteria and Bacteroidetes ranked consistently as the second and third most dominant phyla, respectively. Notably, a greater abundance of Actinobacteria was detected in the skin (9.7% vs. 9.7%) than in the gonad (6.1% vs. 2.9%), gut (3.0% vs. 3.1%), and blood (4.1% vs. 4.8%) in both the female and male groups (Fig. 1e; Fig. S2a; Table S1). At the order level, the mean abundance of *Clostridiales*, belonging to Firmicutes, in the gut (51.9%) was significantly greater than that in the gonad, skin, and blood (28.2%, 18.6%, and 15.8%, respectively). In contrast, a small proportion of *Bacteroidales* (2.3%) was also detected in the gut compared with the gonad, skin, and blood (11.3%, 8.9%, and 18.7%, respectively) (Fig. S3). Although no significant differences were detected among the gonad (9.6%), gut (8.9%), skin (10.4%), and blood (9.0%) samples, we found that the abundance of *Bacillales* was significantly higher in the gonad (12.4%) compensating for the gut (4.4%) in the females compared to the males (2.4% in the gonad and 17.5% in the gut) (Fig. 1f; Fig. S2b). In contrast, the abundance of *Clostridiales* in the testes (39.5%) was higher than that in the ovaries (22.5%), and the opposite trend was detected in the gut (60.5% in females and 34.7% in males). These results suggest a specific compensatory relationship between the gut and gonadal bacterial communities. Regardless of differences between niche and sex, there was no significant difference in the abundance of *Lactobacillales* (Figs. S3 and S4). Previous studies in mammals suggest that internal microbiomes originate from the leakage of gut bacteria into gut-distant organs [7]. SourceTracker2 analyses were performed using the gut, blood, skin, or water as potential sources to determine the source of the gonadal bacterial community. Interestingly, 33.5% of the bacteria in the ovary were of gut origin, whereas 23% were attributable to the blood. In the testis, gut, and blood sources were estimated to represent 31% and 30.5%, respectively. Additionally, we also reported that skin sources accounted for 20% and 13.5% of the ovary and testis, respectively (Fig. 1g). Overall, broad and well-defined ranges of bacteria existed in swamp eel, and notable differences were related to sex.

### Dominant bacteria from the gonads of swamp eel were isolated and cultured

**Fig. 2:**
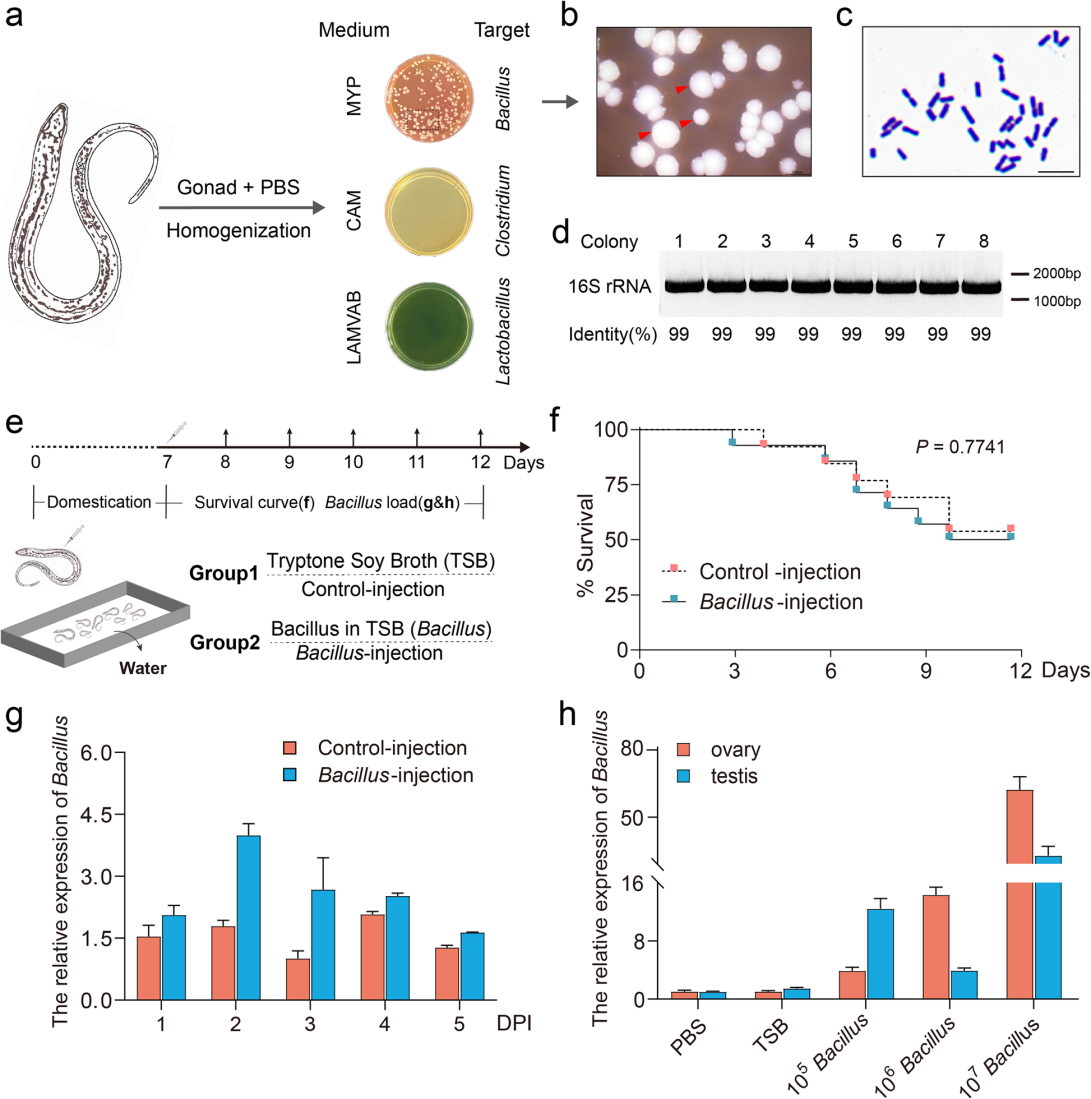
Isolation and injection of dominant bacteria from the ovaries of swamp eel*.* **a** Isolating dominant bacteria from swamp eel gonads using MYP (top), CAM (middle), and LAMVAB (bottom), respectively. **b** Representative colony morphology from MYP medium. Red triangles indicate representative colony morphology. Scale bars, 2000 μm. **c** Representative Gram-stained samples of bacteria from (**b**). Scale bars, 5 μm. **d** Agarose gel electrophoresis of colony PCR samples. The PCR products were verified by agarose gel electrophoresis and sequencing. **e** Schematic diagram of grouping and sampling. Group 1, Tryptone Soy Broth (TSB, control); Group 2, *Bacillus* in TSB (*Bacillus*, treated). **f** Cumulative survival of *Bacillus*-injection fish and the control group. **g** qPCR was applied to detect the *Bacillus* load in swamp eel gonads collected from 1 to 5 days post-injection (DPI). **h** The load of *Bacillus* in the testes and ovaries on day 3 after injection of *Bacillus* at different concentrations. Statistical differences were evaluated by unpaired Student’s *t* test. Data are presented as mean ± SEM of three biological duplicates. *p* values < 0.05 or less were considered statistically significant

Given the unique abundance of bacteria in the gonads, we endeavored to isolate dominant bacteria from the gonads of swamp eel by different selective media. After gonad homogenization and plate coating on selective culture media, we ultimately isolated only round white, gram-positive colonies with neat edges and raised centers, which was highly consistent with the colony morphology of *Bacillus* from MYP medium, with no colonies detected in the other selective media (Fig. 2a–c). Single colonies were subsequently isolated and sequenced via 16S rRNA universal primers. The results revealed that the similarity with *Bacillus* was over 99%, further confirming our successful isolation of *Bacillus* from the ovaries of swamp eel (Fig. 2d; Supplementary file 2).

To evaluate the effects of *Bacillus* on the gonads of swamp eel, we constructed an experimental model in which *Bacillus* was isolated via intraperitoneal injection. When the survival kinetics were analyzed after injection, it was slightly retarded in the *Bacillus* group (50.0%) concerning the control group (53.8%), and no significant difference was detected in these particular groups (Fig. 2e, f). Subsequently, we collected the gonads, including ovaries and testes, and detected the relative expression of *Bacillus.* The results revealed that the load of *Bacillus* in the ovaries increased significantly from day 2 and peaked on day 3 after injection, and then decreased to a level similar to that of the testes (Fig. 2g). Differential *Bacillus* injection concentration gradients also further identified 10^6^ CFU/ml as the sex-specific optimal concentration, i.e., a relatively high load in the ovaries and relatively low load in the testes (Fig. 2h).

### The dominant ovarian bacteria-*Bacillus* contributed to ovarian dysbiosis in swamp eel

**Fig. 3:**
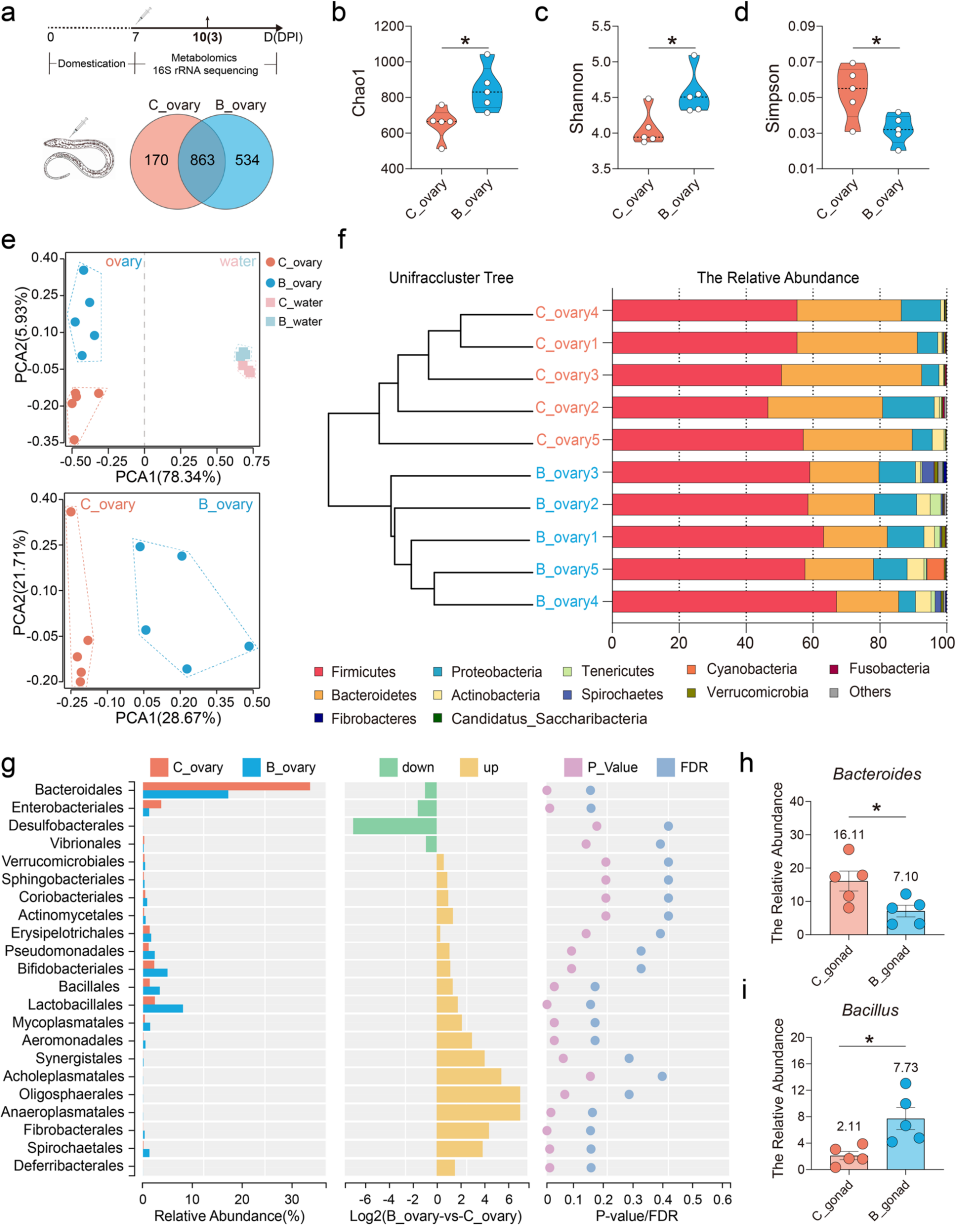
Changes in the microbial distribution in the ovary of swamp eel after *Bacillus* injection. **a** Scheme of the experimental strategy. Ovaries were collected at 3 days post-injection (DPI) for 16S rRNA sequencing and metabolomic analysis. Venn diagram showing the number of the common and unique bacteria in the *Bacillus*-injection and control ovaries. **b**–**d** Richness (Chao1 index) (**b**) and diversity (Shannon (**c**) and Simpson (**d**) index) of the bacterial community in the ovary of control and *Bacillus*-injected fish at 3 days post-injection (DPI). **e** PCA showing the microbiota community from water (top) and swamp eel ovaries (bottom). Samples from the control and *Bacillus*-injected groups were colored with red and blue, respectively. **f** Clustering and composition analysis of the ovarian microbiota at the phylum level after *Bacillus* injection. **g** Differences in microbial communities at the order level. **h, i** Histogram showing the relative abundances of *Bacteroides* (**h**) and *Bacillus* (**i**) in ovaries from the control and *Bacillus*-injected groups. C_ovary, ovaries from the control groups; B_ovary, ovaries from the *Bacillus*-injected groups; C_water, water from the control groups; B_water, water from the *Bacillus*-injected groups. Statistical differences were evaluated by unpaired Student’s *t* test. Data are presented as mean ± SEM of five biological duplicates. *p < 0.05

To assess whether the microbial communities of the ovaries of swamp eel changed after *Bacillus* injection, we performed 16S rRNA sequencing and analyzed the distribution and differences of bacterial communities on day 3 after injection. The culture water was first collected to avoid the impact of the living environment on the microbial community (Fig. S5). After quality filtering and normalization to remove adaptors and low-quality or ambiguous bases, a total of 863 shared OTUs were detected, resulting in 170 and 534 specific OTUs in the control and *Bacillus* injection groups, respectively (Fig. 3a). OTUs were then used to analyze differences in gonadal microbial abundance, and community diversity was compared between the control and *Bacillus*-injected fish. Interestingly, the Chao1 and Shannon indices significantly increased, whereas the Simpson index significantly decreased in ovaries after *Bacillus* injection compared with those in the control group, indicating the *Bacillus* altered the abundance and enhanced the richness of the ovarian bacteria (Fig. 3b–d). We next characterized the similarities or differences in community composition among different groups using the PCA and hierarchical clustering trees. By calculation, we observed that the samples were clustered into two distinct groups associated with variable bacterial communities following *Bacillus* injection (Fig. 3e, f). Specifically, eleven phyla were observed in the ovaries, with Firmicutes, Proteobacteria, Bacteroidetes, and Actinobacteria being the most dominant phyla in both the control (TSB) and *Bacillus* groups. However, Firmicutes (61.1%) were more abundant in ovaries from the *Bacillus* groups compensated by a notable reduction in the abundance of Bacteroidetes (19.7%), compared with the control ovaries (53.0% in Firmicutes and 35.2% in Bacteroidetes) (Fig. 3f). Moreover, we analyzed the bacterial composition changes at the order level. Compared with those in the control fish, the abundances of the orders *Anaeroplasmatales*, *Fibrobacterales*, *Spirochaetales*, *Bacillales*, and *Lactobacillales* were increased in the *Bacillus*-injection fish, whereas the abundances of *Bacteroidales* and *Enterobacteriales* decreased (Fig. 3g). Further analysis revealed that the genera *Bacteroides* and *Bacillus* extensively contributed to the changes in bacterial structure in the control and *Bacillus* groups, respectively (Fig. 3h, i). Additionally, *Bacillus*-induced shifts in short-chain fatty acid (SCFA)-producing bacterial genera, primarily including *Blautia*, *Prevotella*, and *Faecalibacterium* (Fig. S6), suggest potential downstream effects on ovarian metabolic outcomes in swamp eel. Collectively, these data demonstrated that *Bacillus* injection could induce microbial dysbiosis in fish ovarian sites.

### *Bacillus* primarily affected the metabolite profiles in the ovaries, particularly prostaglandin E3

**Fig. 4:**
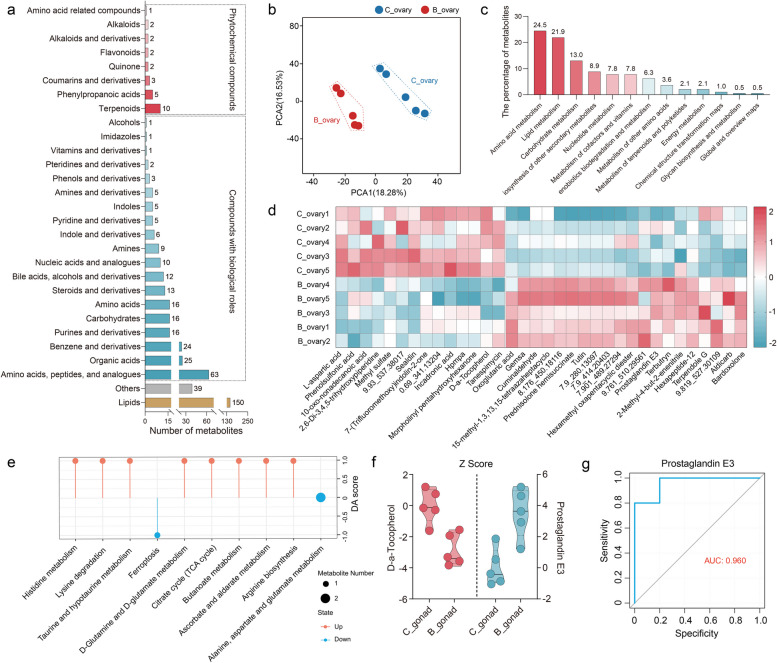
Metabolic alterations of the ovary induced by *Bacillus* in swamp eel. **a** The bar chart displays the classification and content of identified metabolites. **b** PLS-DA representing the ovarian metabolite profiles from the control and *Bacillus*-injected groups. **c** Classification of metabolic pathways in the ovaries of swamp eel*.* **d** Content heatmap of differential metabolites identified in the control and *Bacillus*-injected ovaries. The numbers (e.g., 9.93_537.38017) represent unidentified metabolites. **e** Differential abundance scores from the metabolic pathway enrichment analysis. **f** The relative contents of D-α-Tocopherol and prostaglandin E3 were converted and measured with Z-score. **g** ROC curve analysis of prostaglandin E3 for screening potential biomarkers. The AUC value is a measure of the ability of a metabolite to act as a biomarker. C_ovary, ovaries from the control groups; B_ovary, ovaries from the *Bacillus*-injected groups

To further explore the differences in metabolites underlying the *Bacillus* of ovaries in female swamp eel, we performed full-spectrum and non-targeted metabolomic analysis and identified a total of 2906 metabolites across ovary samples in swamp eel. Lipids were the most abundant category of compounds in the ovaries, followed by phytochemical compounds (amino acids, peptides and analogues, organic acids, benzene and derivatives, etc.,) and compounds with biological roles (terpenoids and phenylpropanoic acid, etc.,) (Fig. 4a). PLS-DA analysis was performed to determine the overall inter- and intra-group differences in the metabolomes between the control and *Bacillus*-injected ovaries. We found that the samples did not overlap and were well distinguished from each other between the two groups, suggesting that *Bacillus* could alter metabolite profiles in the ovaries (Fig. 4b). Subsequently, we analyzed the differential metabolites leading to sample separation. By comparing ovarian metabolites between the control and *Bacillus* injection groups, we found that the differential metabolites were enriched mainly in the amino acid metabolism (24.5%), lipid metabolism (21.9%), and carbohydrate metabolism (13.0%) (Fig. 4c). Compared with those in the control group, the concentrations of prostaglandin E3, oxoglutaric acid, terbutryn, prednisolone hemisuccinate, bardoxolone, cuminaldehyde, and tutin were significantly higher in the *Bacillus* injection group, whereas the concentrations of the other ovarian metabolites, including tanespimycin, incadronic acid, methyl sulfate, hpmpa, sealdin, l-aspartic acid, and d-α-Tocopherol, were significantly lower in the *Bacillus* injection group (Fig. 4d). Differential abundance score analysis was performed to determine the overall changes in the differential pathways. Compared with those in the control group, the functional pathways related to metabolic pathways, especially histidine metabolism, taurine and hypotaurine metabolism, d-glutamine and d-glutamate metabolism, and butanoate metabolism were significantly enriched in the *Bacillus* injection group. In contrast, the control group exhibited markedly higher enrichment in ovarian pathways associated with ferroptosis. Although the functional pathways involved in alanine, aspartate and glutamate metabolism tended to be enriched in the ovaries of the control group, there were no significant differences between the two groups (Fig. 4e). The Z-score was then calculated to measure the relative amounts of metabolites at the same level. The results revealed d-α-Tocopherol was downregulated, whereas prostaglandin E3 was upregulated in the ovaries of the *Bacillus* injection group compared to the control group (Fig. 4f). Moreover, the ROC curve analysis revealed that prostaglandin E3 may serve as a potential biomarker after *Bacillus* injection (Fig. 4g). Overall, the metabolic components of the ovary were altered after *Bacillus* injection in swamp eel.

### l-aspartic acid refracted microbial and metabolic alterations governed by *Bacillus* injection

**Fig. 5:**
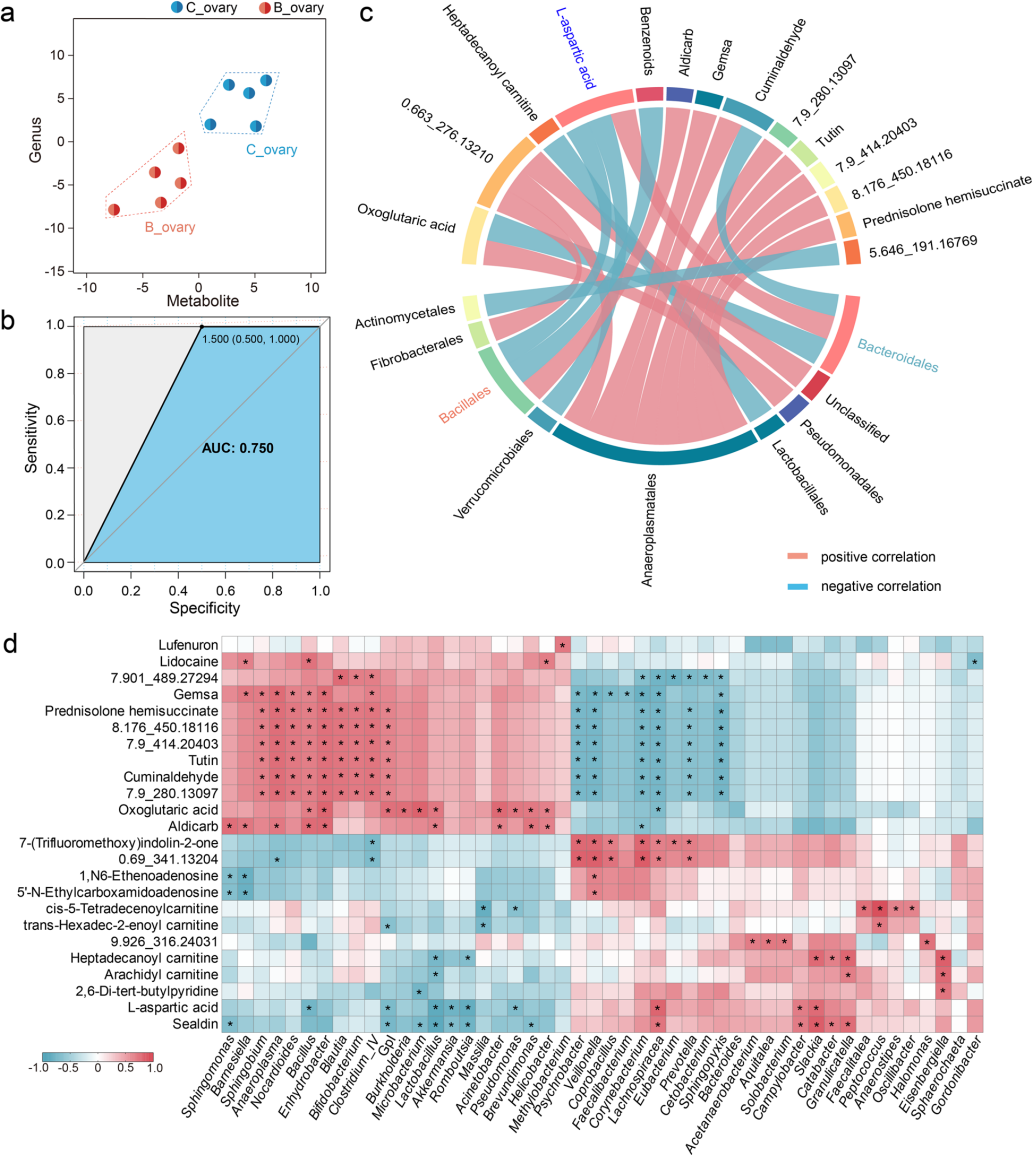
Correlation analysis of differential bacteria and altered metabolites in swamp eel. **a** Combining differential microbial communities and metabolite composition for PCA analysis. **b** Screening microbial communities and metabolic biomarkers for distinguishing biological phenotypes.** c** Spearman correlation chord diagram of microbial groups and differential metabolites. The connecting line in the circle represents the correlation between microbial groups and metabolites; red represents a positive correlation and blue represents a negative correlation. The numbers (e.g., 7.9_280.13097) represent unidentified metabolites. **d** Heatmap showing the correlation analysis between the differential microbes and the differential metabolites in the ovaries. The correlation coefficient is represented by different colors: red represents a positive correlation, and blue represents a negative correlation. The numbers (e.g., 7.901_489.27294) represent unidentified metabolites. *Represents significantly negative or positive correlations: *p < 0.05

Correlation analysis of 16S amplicon sequencing and metabolomics was performed to explore the relationship between the ovarian microbiota and host metabolism using the data of differential microbiota and metabolites. According to the PCA results, all five samples in each group clustered together, further indicating that the ovarian samples were altered in each group after *Bacillus* injection (Fig. 5a). In addition, ROC curve analysis revealed an AUC value of 0.75 (> 0.5), which confirmed the reliability of the data (Fig. 5b). Spearman’s correlation analysis of the microbial species analysis also indicated some patterns of specific associations between bacteria and metabolites in swamp eel (Fig. 5c, d)*.* For example, the relative abundance of the order *Bacillales* in the *Bacillus* group was higher, which was positively correlated with aidicarb but negatively correlated with l-aspartic acid. Conversely, the lower abundance of *Bacteroidales* was positively correlated with l-aspartic acid but negatively correlated with oxoglutaric acid and cuminaldehyde (Fig. 5c). A heatmap of the genus-level microbial species analysis also revealed more relevant expression patterns. l-aspartic acid was positively correlated with *Lachnospiracea* and *Bacteroides* but negatively correlated with *Bacillus*, *Lactobacillus*, and *Romboutsia*. Moreover, *Bacillus* was positively correlated with lidocaine, gemsa, and aldicarb (Fig. 5d).

### *Bacillus* inhibited testicular function in hermaphroditic swamp eel

**Fig. 6:**
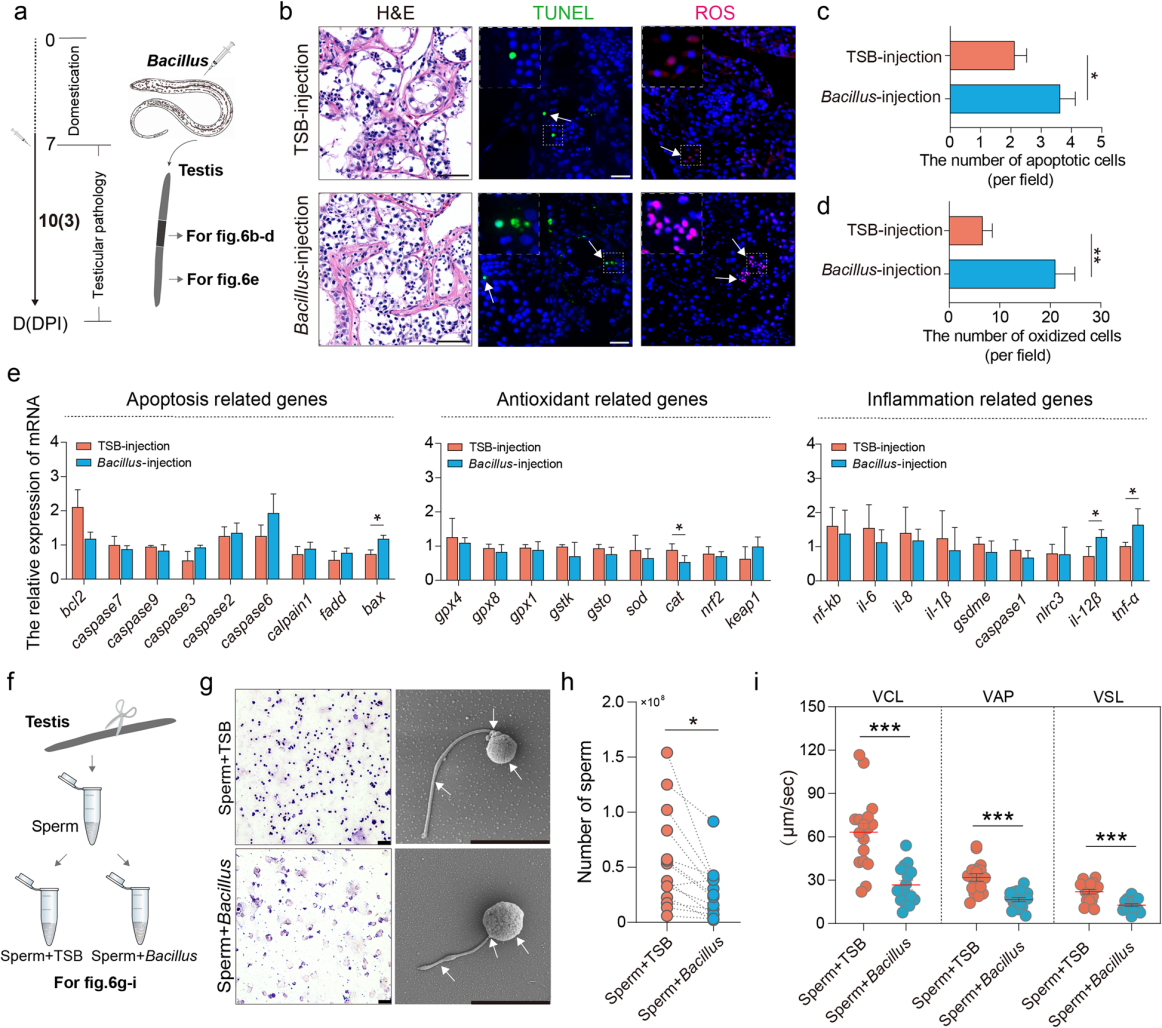
*Bacillus* led to testicular inflammatory responses accompanied by decreased sperm motility in male swamp eel*.* **a** Experimental design schematic for the testes. **b** Representative H&E (left), TUNEL (middle), and DHE (right) staining of testes from control and *Bacillus*-injected swamp eel. The white arrowheads indicate TUNEL-positive and DHE-positive cells, respectively. The white dashed boxes represent the enlarged area image. Scale bars, 20 μm. **c, d** Counts of TUNEL-positive (**c**) and DHE-positive cells (**d**) in testes from control and *Bacillus*-injected swamp eel*.* **e** Expression of apoptosis-, antioxidant-, and inflammation-related genes in the swamp eel testes after treatment with *Bacillus*. **f** Experimental design schematic for the sperm. **g** Representative Giemsa staining and TEM images showing the sperm morphology after incubation with *Bacillus.* The white arrowheads indicate altered sperm morphology in the head, midpiece, and flagellum. Scale bars represent 40 μm and 5 μm for Giemsa staining and TEM, respectively. **h** The number of sperm from the control and *Bacillus*-incubated groups. **i** Sperm motility analysis including VCL, VAP, and VSL showing the variability in sperm quality between the different groups. Statistical differences were evaluated by unpaired Student’s *t* test. Data are presented as mean ± SEM of three biological duplicates. *p < 0.05, **p < 0.01, ***p < 0.001

Based on the results that ovarian dominant *Bacillus* altered ovarian microbes and metabolites, we further explored the effects on the ovary-testis transmission. Histological sections revealed no significant changes in the morphology of the testis after *Bacillus* injection. However, apoptosis was induced along with a prominent decrease in antioxidant levels, as shown by TUNEL and ROS staining, respectively (Fig. 6a–d). Several genes, including antioxidant-related genes, apoptosis-related genes, and inflammation-related genes were then quantified in testis samples via RT-qPCR, and the results were similar. As expected, the *Bacillus* injection group presented significantly higher expression of *keap1* and lower expression of *cat* and *sod* than the control group. In addition, apoptosis-related genes showed significantly higher expression, especially *bax*. Importantly, the inflammation-related genes *tnf-α* and *il-12* were also significantly upregulated, further suggesting a concomitant inflammatory response (Fig. 6e). To further investigate the sperm response to *Bacillus*, we isolated sperm from swamp eel and incubated them with *Bacillus*, and found that the sperm morphology was significantly altered and the sperm concentration was significantly reduced (Fig. 6f–h). When the sperm motility was analyzed, our results indicated that *Bacillus* significantly decreased all motility parameters, reducing the average path velocity (VAP), linear velocity (VSL), and curvilinear velocity (VCL) (Fig. 6i). The above results indicated that the dominant bacteria in the ovaries induced inflammation in the testes, accompanied by a decrease in sperm motility.

## Discussion

Microorganisms contribute to gonadal function and reproductive health, with the reproductive tract of the ovary being the best characterized [1–3]. To date, the gut microbiota has been widely studied, but direct evidence for microbial communities in healthy gonads has not been proposed, particularly in the testis. Fish, in direct and constant contact with their aquatic environment, host high densities of microbiota that interact with them and play crucial roles in growth enhancement, nutrition, development, and immunity [20]. Understanding the fish microbiota is essential for identifying both beneficial and dysbiotic phenotypes [21]. Our findings revealed significant associations between the bacteria and gonads during healthy physiological states in a hermaphroditic species of swamp eel, with over 55% of their bacterial diversity shared with that found in the gut and blood. It remains to be investigated whether this phenomenon is characteristic of other teleosts or a universal symbiotic relationship present in all vertebrates.

Our study provides several potential mechanisms by which bacteria can colonize the swamp eel gonads. First, our data suggest that gonad bacteria are partly sourced from the gut and the blood and that bacteria appear to cross the blood-testis/gonad barrier and colonize the parenchyma in healthy swamp eel without causing any disease. It is considered that dominant bacteria may reside in different stages of gonadal transmission during natural sex reversal in swamp eel. However, our attempts to inject gonadal dominant bacteria into the ovaries of female swamp eel and cultivate them to observe the sex reversal process were not successful. Therefore, we continue our efforts to isolate the gonadal dominant bacteria and investigate their functions in the ovaries (Figs. 3, 4, and 5) and testes (Fig. 6) respectively. Recent studies have indicated that various body niches, such as the upper female gynecological tract and blood, can no longer be considered germ-free under healthy conditions due to the presence of commensal or native microbial flora, collectively referred to as the microbiome [7, 22–25]. It was previously thought that bacteria had difficulty crossing the blood–brain barrier and the blood-testis barrier. However, microorganisms have now been identified in both diseased brains and gonads. Notably, bacteria appear to cross the blood–brain barrier in healthy rainbow trout without causing any disease [7]. In mammals, the microbiota has been found to reside within phagocytes [26]. These phagocytes may act as reservoirs for various bacteria capable of crossing either the blood–brain or the blood-testis barriers, which may help explain our findings of gonadal dominant bacteria in both the ovaries and testes. We aim to explore this question more thoroughly in future studies.

Sex differentiation and gonadal plasticity exhibit remarkable diversity among vertebrate species [27]. However, unlike the XY female syndrome observed in humans [28], fish exhibit a natural phenomenon of sex reversal [9]. The environment and genes interact in many species to determine sexual fate and regulate gonadal development, with classic environmental influences including temperature [29]. Our work expands our understanding of the environmental factors that regulate gonadal development, including those related to microorganisms within gonadal tissue. In our case of hermaphroditic swamp eel, both the ovarian and testis microbial communities maintain homeostasis. Specifically, ovarian-dominant bacteria play crucial roles in supporting ovarian function; for instance, *Bacillus* significantly influences metabolite profiles, particularly prostaglandin E3, which contributes to ovarian dysbiosis. Interestingly, *Bacillus* have been widely used as environmental probiotics and dietary probiotics in aquaculture because they produce anti-microbial substances and enzymes that provide their ability to colonize the digestive tract and contribute to the nutrition of the host [30]. In addition, *Bacillus* treatment has been reported to alter the structure and composition of the fish gut microbiota [31, 32]. These findings also suggest that the homeostasis of gonadal microbial communities is influenced by feeding, with most microorganisms originating from the gut microbiota (Fig. 1g). Growing evidence suggests that the Firmicutes/Bacteroidetes ratio in the gut is also associated with polycystic ovary syndrome (PCOS) [33–35]. A critical unanswered question is how this ratio in the gut influences ovarian function. Several studies have proposed that gut microbiota metabolism can have direct effects on the intestines or enter the systemic circulation, thereby affecting host tissues, including the ovaries [34, 35]. Our study establishes direct communication between the gut and gonadal microbial communities and redefines the physiological relationships between gonads and bacteria in teleosts.

Microorganisms commonly influence tissue function by producing metabolites and disrupting the balance of special metabolic or immune-metabolic interactions [16, 19, 36]. Consequently, we concentrated on the interactions of microbial components within the ovaries following *Bacillus* injection (Fig. 3) and analyzed the changes in ovarian metabolites post-injection using mass spectrometry (Fig. 4). Our results revealed a significant increase in the Firmicutes/Bacteroidetes ratio in the ovaries after *Bacillus* injection (Fig. 3f), a trend that was also noted when *Bacillus* was added to the zebrafish diet [37]. Furthermore, lipid metabolism and amino acid metabolism were the two metabolic pathways most profoundly affected by *Bacillus* injection in the ovaries (Fig. 4c). We observed an increase in the differential metabolite prostaglandin E3, whereas d-α-tocopherol levels decreased in the *Bacillus*-injection group. Prostaglandins are crucial mediators of various reproductive processes and are essential for ovulation in all vertebrate species [38]. Prostaglandin E2, known for its importance in mammalian ovulation, also plays a role in the ovulatory process in several teleosts, including medaka and zebrafish [39, 40]. Given the shared E-type prostanoid receptor system between prostaglandin E2 and E3 [41], the increased levels of ovarian prostaglandin E3 resulting from *Bacillus* injection may enhance the subsequent ovulation process in swamp eel. Interestingly, l-aspartic acid appeared to be a distinct differential metabolite in the ovaries of swamp eel and was associated with the dominant ovarian bacteria (*Bacillales* and *Bacteroidales*). Generally considered an antioxidant, aspartic acid helps maintain the integrity of reproductive organs, particularly the testes and epididymis [42]. Increasing evidence suggests that metabolites are linked to bacterial dysbiosis in teleosts, highlighting the strong association between specific bacteria and metabolites [43–45]. Our findings suggest that l-aspartic acid may be present as a candidate indicator of bacterial abundance and reproductive health.

A critical question arising from our work is how gonadal microbial communities are involved in female-to-male sex reversal in swamp eel. Some limitations of this study include our inability to track changes in the microbiome during this female-to-male transition after the injection of ovarian-dominant bacteria, such as *Bacillus*. Natural sex reversal often lasts more than 8 months, making it difficult for swamp eel to survive for such an extended period following injection. As a result, it remains unclear whether gonadal microbial communities influence natural sex reversal or vice versa. Understanding the causal relationship between these two could illuminate an unresolved mystery that has persisted for over 80 years regarding the factors that drive natural sex reversal. Our research demonstrated that ovarian-dominant bacteria, such as *Bacillus*, play dual roles in both the ovaries and testes of hermaphroditic animals. These findings provide in vivo evidence that changes in gonadal microbial communities can affect gonadal function during sex reversal.

Different reproductive environments present significantly different bacterial communities, which is critical for sex-specific differences in gene expression and metabolism in mammals [6, 46]. It has been demonstrated that the vaginal probiotic *Lactobacillus* greatly affected sperm activity and showed a high adhesion to sperm in mammals [47]. Previous studies have indicated that altered testicular microbiome composition is associated with a highly inflammatory state in zebrafish testes [18]. The blood-testis barrier plays a critical role in maintaining the microenvironment essential for the function of the testes but triggers an inflammatory response in the testes when it is disrupted [48]. Unlike NF-κB, which prevents the transition from juvenile ovaries to testes in zebrafish through anti-apoptotic signaling [49, 50], the injection of *Bacillus* resulted in no significant change in *NF-κB* expression or several apoptosis-related genes in swamp eel testes, except for *bax*. Among the inflammation-related genes, only *tnf-α* and *il-12β* were significantly activated, while the oxidative stress-related gene *cat* was significantly repressed by *Bacillus* (Fig. 6e).

Apoptosis, inflammation, and oxidative stress can impair sperm function [51], leading us to further investigate the effects of *Bacillus* on sperm function. Sperm motility is a key factor in assessing sperm function and predicting fertilization potential [52]. Due to the complex seminal vesicle structure of the swamp eel testis, the sperm is usually collected by dissecting the testes, cutting them into pieces, and homogenizing rather than by squeezing the abdomen like many other fishes. The incorporation of gonadal cells and immature sperm can cause an underestimation of activated sperm and the offspring yield. Although the sperm activity in swamp eel is suggested to be low, activated sperm could maintain movement for a long time with a relatively long lifespan [53]. Our study revealed that *Bacillus* not only significantly inhibited sperm motility but also reduced the sperm count in swamp eel (Fig. 6h, i). Mechanistically, this may be related to increased apoptosis induced by upregulated expression of the *bax* gene, inflammatory pathways activated by *tnf-α* and *il-12β*, and oxidative stress resulting from decreased expression of the *cat* gene by *Bacillus*. Therefore, artificial injection of ovarian-dominant bacteria, such as *Bacillus*, likely disrupts the physiological homeostasis of microorganisms and subsequently induces inflammation and an oxidative response in the testes. This finding also suggests that when environmental and dietary probiotics are added to aquaculture, their potential impact on aquaculture reproduction should be carefully considered, even including how human consumption of probiotics might affect gonadal function.

## Conclusions

In summary, our findings demonstrate that microorganisms directly colonize the gonads and underscore their essential role in relevant metabolic pathways within the gonads of swamp eel*.* Specifically, gonadal microbial communities at homeostasis are comparable to those found in other distinct niches. After injection with the ovarian dominant bacteria *Bacillus*, the abundance of ovarian microbiota involved in amino acid and lipid metabolism is significantly altered, possibly contributing to the occurrence of ovarian function in swamp eel*.* In contrast, *Bacillus* induces testicular inflammation and reduces sperm motility in male swamp eel. Based on the above results, our study not only redefines the boundaries between healthy vertebrate gonads and microbiota but also opens new avenues for research on microbiota-driven modulation of gonadal homeostasis. Further studies will investigate the role of bacterial microbiota during sex reversal in swamp eel.

## Materials and methods

### Fish maintenance and trial design

The sourcing of swamp eel and the rearing procedures employed in this study have been described in previous studies [6, 54]. Briefly, healthy and mature individuals (with females weighing 80–150 g and males > 250 g) from Baishazhou Aquatic Products Wholesale Market (Wuhan, China) were selected as parents to generate the sibling offspring via artificial insemination. The embryos and larval swamp eel were incubated in the incubator of a cell culture room for 4 months, reaching the juvenile stage at 120 days post-fertilization (dpf). The juveniles were then transferred to the indoor recirculating system, maintained at a temperature of 25–28 °C, a pH of 7.2–8.0, and adequate dissolved oxygen levels. They were fed with frozen bloodworms at a rate of 0.5–1% of body weight 2–4 times daily for an additional 10 months, with feeding ceasing 7 days prior to sacrifice. Finally, adult swamp eel was sampled at 430 dpf for subsequent investigations.

Before sampling, the water in which the fish lived was first collected for 16S rRNA sequencing to obtain the background composition of the bacterial community in the water environment. Subsequently, the swamp eel was anesthetized with MS-222, and the blood was collected using a disposable sterile syringe. After sterile dissection of the skin and gut, the gonads were separated and divided into two segments. One of them was fixed immediately in 4% neutral formalin buffer for sex identification and histological observation. The other segment was stored in sterile freezing tubes for sequencing. All of these collected tissues were immediately frozen with liquid nitrogen and stored at − 80 °C for further study.

### Histology and light microscopy studies

After being fixed in 4% neutral formalin buffer at 4 °C overnight, gonadal samples extracted from female and male swamp eel were dehydrated in a graded ethanol series, permeabilized with xylene, embedded in paraffin, and then cut into 4 μm sections. The sections were rehydrated and stained with hematoxylin and eosin (H&E) for routine histological examination as follows: dewaxed in xylene, rehydrated through a graded ethanol series, stained the nucleus with a hematoxylin solution, and the cytoplasm with an eosin solution, finally sealed with resin. Images were collected and analyzed using an automatic digital slide scanner (Leica, Germany).

### Identification and challenge of gonadal bacteria in swamp eel

To verify the key bacteria in the gonads, we sampled the gonadal tissue of swamp eel in sterile PBS. After homogenization by bead beating for 2 min at 60 Hz, 100 μl homogenate was absorbed onto the plate preparation of Mannitol-Egg-Yolk-Polymyxin Agar Base (MYP), Columbia Agar Medium (CAM), and LAMVAB Agar (LAMVAB), respectively. After being incubated upside-down for nearly 14 h at 37 °C in a humidified CO₂ incubator, single colony was then selected and cultured in Brain Heart Infusion Broth (BHI) following 12h incubation at 37 °C with continuous shaking (220 rpm) in a temperature-controlled incubator. Bacterial universal primers were used for amplification in a 50-μl reaction, including 25 μl 2 × PCR master mix, 2 μl bacteria template, and 2 μl primers as the following conditions: 95 °C for 10 min, followed by 35 cycles of 95 °C for 30 s, 58 °C for 30 s, 72 °C for 1 min and a final extension at 72 °C for 10 min. After being analyzed by agarose gel electrophoresis and photographed, the PCR products were purified and sequenced.

For the swamp eel challenge, we made slight modifications according to previous studies [55, 56]. Specifically, 60 fish (∼ 100–120 g) were challenged with *Bacillus* via intraperitoneal injection at a final concentration of 10^5^, 10^6^, and 10^7^ CFU/ml for each challenge experiment, and then migrated into the aquarium containing fresh culture water. Gonads were collected daily from 3 individuals from days 1 to 5 after injection. Both experiments were performed at least three independent times. As a control, the same number of fish were maintained in a similar tank and injected with the same culture medium without bacteria. The samples from the control fish were also collected from days 1 to 5 after the medium culture mock challenge. The gonads from 10^6^ CFU/ml challenged female swamp eel were collected for subsequent 16S rRNA gene sequencing and metabolome sequencing. The testes challenged with the same concentration were collected for downstream research. Moreover, the water in which the fish lived was also collected for 16S rRNA sequencing before sampling.

### RNA isolation and quantitative real-time PCR analysis

Total RNA was extracted according to the manufacturer’s instructions. The concentration of RNA was measured using a spectrophotometer (Nanodrop One), and the completeness was analyzed by agarose gel electrophoresis. Approximately 2 μl of RNA (1000 ng in total) was reverse transcribed into cDNA with Hifair III First-Strand cDNA Synthesis SuperMix for qPCR (YEASEN) following the manufacturer’s instructions. The synthesized cDNA was diluted five times and then used as a template for quantitative real-time PCR (qRT-PCR) analysis. The qRT-PCR was performed in triplicate, and each contained 1 μl of a diluted cDNA template, 5 μl of SYBR Real-time PCR Master Mix plus (Monad), and 0.5 μl forward and reverse primers in a 10-μl reaction volume. The amplification profile was performed as follows: 95 °C for 2 min, followed by 40 cycles of 95 °C for 10 s, 60 °C for 10 s, and 72 °C for 10 s. A dissociation protocol was carried out by reading fluorescence at every 0.5° between 72 and 95 °C after thermal cycling to confirm that only a single band of the correct size was amplified. The relative expression levels were analyzed by the 2^−∆∆CT^ method, and elongation factor 1α (*ef-1α*) and ribosomal protein L17 (*rpl17*) were used as internal controls [6, 57]. The primers used in this study are listed in Table S2.

### DNA extraction, PCR amplification, and Miseq sequencing

The genomic DNA was extracted using the MagPure Stool DNA KF Kit B (Magen, China) following the manufacturer’s instructions. The concentration and quality of the extracted DNA were subsequently determined with a Qubit fluorometer (Invitrogen, USA) and checked by running an aliquot on a 1% agarose gel, respectively. PCR amplification was performed in a 50 μl reaction mixture to obtain the V3–V4 regions of the 16S rRNA gene using 30 ng template, PCR master mix, and the bacterial universal primers 338F (5′-ACTCCTACGGGAGGCAGCAG-3′) and 806R (5′-GGACTACHVGGGTWTCTAAT-3′) as the following program: 94 °C for 3 min, followed by 30 cycles of 94 °C for 30 s, 50 °C for 45 s, 72 °C for 45 s, and final extension at 72 °C for 10 min. The PCR products were purified with Agencourt Ampure XP beads and eluted in elution buffer. Libraries were qualified by the Agilent 2100 bioanalyzer (Agilent, USA). The validated libraries were applied for sequencing on the Illumina MiSeq platform (BGI, China) following the standard pipelines of Illumina, and generating 2 × 300 bp paired-end reads.

Raw reads were filtered by an in-house procedure and paired-end reads were merged to tags with FLASH [58]. The tags were clustered into OTUs with a 97% threshold by using UPARSE, and the OTUs unique representative sequences were obtained. Then, OTUs representative sequences were taxonomically classified using the RDP Classifier with a minimum confidence threshold of 0.6, and trained on the RDP Release16 database by QIIME2 [59]. The USEARCH global was used to compare all tags back to OTUs to obtain the OTU abundance statistics table of each sample. Alpha and beta diversity were estimated by MOTHUR and QIIME2 at the OTU level, respectively. PCA of the OTUs was plotted with the R package “ade4.” The relative contribution of microbial communities was predicted by microbial Sourcetracker2 analysis [60]. LEfSe cluster or LDA analysis was conducted by LEfSe. The sample clustering was conducted by QIIME2 based on UPGMA. Barplot and heatmap of different classification levels were plotted with R package v3.4.1 and R package “gplots,” respectively.

### Metabolite extraction and LC–MS conditions for analysis

The samples (approximately 25 mg) were employed for metabolite extraction by mixing with 800 μl precooled extraction reagent (methanol: acetonitrile: water (2:2:1, v/v/v)) and lysis using a TissueLyse bead-mill homogenizer (Qiagen, Germany). After centrifugation at 25,000 g at 4 °C for 20 min, 600 μl of supernatant from each sample was collected and filtered using an SPE column for solid-phase extraction, and the soluble metabolites were dissolved in acetonitrile. Approximately 10 μl from each sample was pooled together as a QC sample and used to assess the reproducibility and reliability of the LC–MS method.

Subsequently, chromatographic separations were performed using an ultra-performance liquid chromatography (UPLC) system (Waters, USA). An ACQUITY UPLC BEH C18 column (100 × 2.1 mm, 1.7 μm; Waters, USA) was used for the reversed phase separation. The column oven was maintained at 45 °C. The flow rate was set at 0.35 ml/min, and the mobile phase consisted of 0.1% formic acid (A) and acetonitrile (B) in the positive mode, and in the negative mode, the mobile phase consisted of 10 mM ammonium formate (A) and acetonitrile (B). The eluted metabolites were introduced into a Q Exactive HF high-resolution mass spectrometer (Thermo Fisher Scientific, USA). The full scan range was 70 to 1050 m/z with a resolution of 120,000. The top 3 precursors were selected for subsequent MS fragmentation with a maximum ion injection time of 50 ms and a resolution of 30,000. The stepped normalized collision energy was set to 20, 40, and 60 eV. Electrospray ionization (ESI) parameters were set as: sheath gas flow rate was 40, Aux gas flow rate was 10, positive-ion mode spray voltage was 3.8 kV, negative-ion mode spray voltage was 3.2 kV, capillary temperature was 320 °C, and Aux gas heater temperature was 350 °C.

The raw data were imported into the software Compound Discoverer 3.3 for peak detection and alignment. The assigned modified metabolites were identified by searching the HMDB database (http://www.hmdb.ca/spectra/ms/search) using Progenesis QI. The molecular mass data (m/z) and retention times (min) were used to identify the metabolites. The putative metabolites were mapped to the Kyoto Encyclopedia of Genes and Genomes (KEGG; http://www.genome.jp/kegg/) by matching the m/z of our samples with those from the database. The pre-processed data were imported to MetaX, an R package software for statistical analysis. The dataset was also subjected to unsupervised multivariate statistical analyses, including principal component analysis (PCA) and partial least-square discriminant analysis (PLS-DA), using SIMCA v.13.0 software. Responsive metabolites were identified using receiver-operating characteristic (ROC) curve analysis by calculating the area under the curve (AUC) using the pROC package in R. Cytoscape v.3.3 was used for visualization of the relationship among metabolites and pathways.

### Microbiome‑metabolome integrative analysis

For the association analysis, correlation coefficients were calculated using the abundance of metabolites and the relative abundance of microbial groups. Data were neutralized and normalized. Following unsupervised analyses on each dataset, which were completed to explore and visualize any changes according to treatments, the integration was carried out. In addition to the PCA and PLS-DA, which represented the overall situation of the original data, the heatmap produced correlations between the microbial groups and differential metabolites. By comparing the ROC curves among the separate modeling of different omics and the combined data modeling, which omics can better screen the best biomarkers for the two groups was evaluated. Moreover, Spearman’s correlation analysis evaluating the monotonic relationship between two continuous or ordinal variables was performed using the corr.test in R software.

### Testis DHE and TUNEL staining

Changes in ROS production induced by *Bacillus* were evaluated by staining with dihydroethidium (DHE, Beyotime). Briefly, testis sections were paraffined, dewaxed with xylene, washed with ethanol, and then incubated with DHE at a final concentration of 10 mM at 37 °C for 40 min in the dark. After being washed with PBS, the sections were stained with 1 μg/ml 4,6-diamidino-2-phenylindole (DAPI) for 10 min in the dark at room temperature. The fluorescence was observed under an Olympus IX51 fluorescence microscope.

TUNEL staining of tissue sections was conducted using the In Situ Cell Death Detection Kit (Roche, 11,684,817,910). Briefly, testis sections were paraffined, dewaxed with xylene, washed with ethanol, and then incubated with proteinase K at 37 °C for 30 min, followed by incubation with the provided fluorescein-conjugated TUNEL reaction mixture in a humidified chamber at 37 °C for 2 h in the dark. The cell nuclei were further stained with 1 μg/ml DAPI for 10 min in the dark at room temperature. The fluorescence was observed under an Olympus IX51 fluorescence microscope.

### Sperm collection and SEM scanning

The testis of each male swamp eel was removed and squeezed with tweezers for the preparation of the sperm sample, which was temporarily kept in ice-cold Hank’s Balanced Salt Solution (HBSS) as previously described [53]. Subsequently, the sperm from each species were plated on poly-d-lysine-coated cover glasses and submerged into fixation solution overnight at 4 °C. Next, the sperm were postfixed in 1% osmium tetroxide in 0.1 M phosphate buffer (PB) for 2 h in the dark and sequentially dehydrated in 30, 50, 70, 80, 90, 95, 100, and 100% ethanol solutions for 15 min. The samples were critical point dried with liquid carbon dioxide (Quorum, China), mounted on aluminum stubs, and metalized with gold (Hitachi, Japan) for later observation using a scanning electron microscope (Hitachi, Japan).

### Sperm motility analysis

Taking 50 μl purified sperm samples were mixed with BHI medium containing *Bacillus* in equal volumes (the ratio of sperm to bacteria was 1:10), and the mixed samples were incubated at room temperature for 4 h, with slight modifications to the previous report [52]. Subsequently, a mixture of 10 μl was placed on a slide to dry, then fixed with methanol for 30 min, stained using the Gram staining procedure, and examined under an optical microscope (Olympus BX63). Moreover, after being diluted to an appropriate concentration, a 0.5 μl sperm sample was added to 2 μl water to activate the sperm on a specialized slide glass (2 × cells, 20 μm, Hamilton Thorne) and covered with a matched coverslip. A computer-assisted sperm analysis system (CEROS II Animal, Hamilton Thorne, USA) was applied to detect and analyze sperm motility after incubation with the tested bacteria for 4 h. At least eight regions of the chamber were recorded for each sample, and the entire experimental procedure starting from the dissected testis was completed within 6 h for all samples. The sperm parameters related to total motility were measured and summarized in Table S3.

### Statistical analysis

The unpaired Student’s two-tailed *t* test (Prism version 8.0; GraphPad) was performed to determine the statistical significance in the bacterial diversity and abundance, gene expression, testis histology, and sperm motility. *P* values of 0.05 or less were considered statistically significant.

## Supplementary Information

Supplementary File 1.

## Data Availability

Sequence data that support the findings of this study have been deposited in the NCBI Sequence Read Archive under BioProject accession numbers PRJNA1101748 and PRJNA1101906, and be provided within the manuscript.
