# Supplementary material for: Natural sex reversal imparts permanent compositional changes to the swamp eel gonadal microbiome
Source: Microbiome. 2025 Oct 24;13:217. doi: 10.1186/s40168-025-02187-7 (PMC12551287; doi:10.1186/s40168-025-02187-7)
Supplement: Supplementary file 2 — Supplementary File 1. [file 40168_2025_2187_MOESM1_ESM.docx]

**Supplementary Information**

Natural sex reversal imparts permanent compositional changes to the swamp eel gonadal microbiome

Kaifeng Meng, Meidi Hu, Yuanyuan Chen, Xing Lin, Chaoling Jiang, Jiarui Song, Yifan Bai, Yuanli Zhao, Fei Liu & Daji Luo


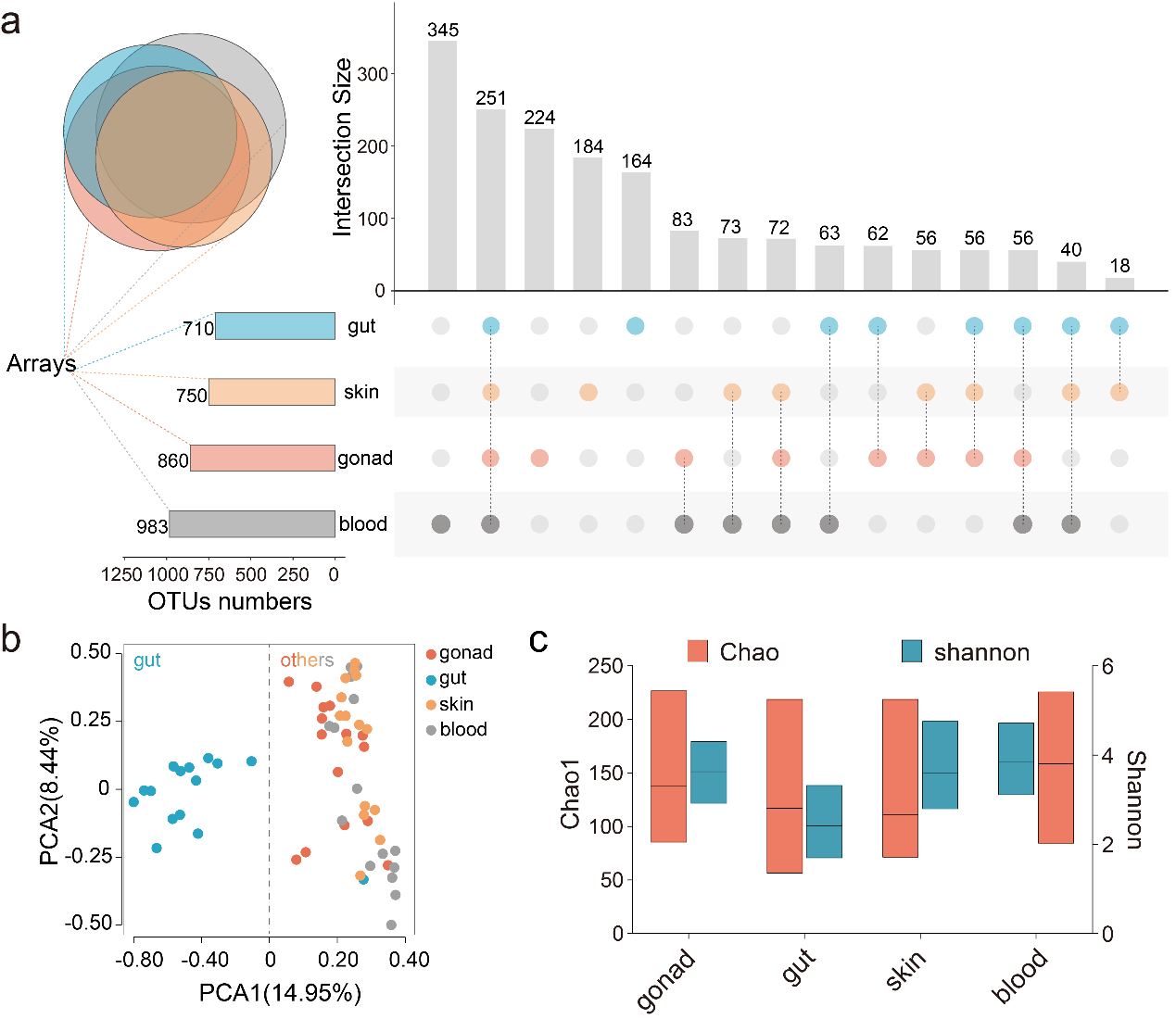


**Figure S1.** Swamp eel exhibits distinct bacterial characteristics in different niches. **a** The Venn diagram and upset plot of the microbiota in the gonad, gut, skin, and blood. **b** PCA showing the microbiota community from the gonad, gut, skin, and blood of swamp eel. **c** Richness and diversity of the bacterial community in the gonad, gut, skin, and blood of swamp eel. Community richness and diversity were measured by the Chao1 index and Shannon index, respectively.


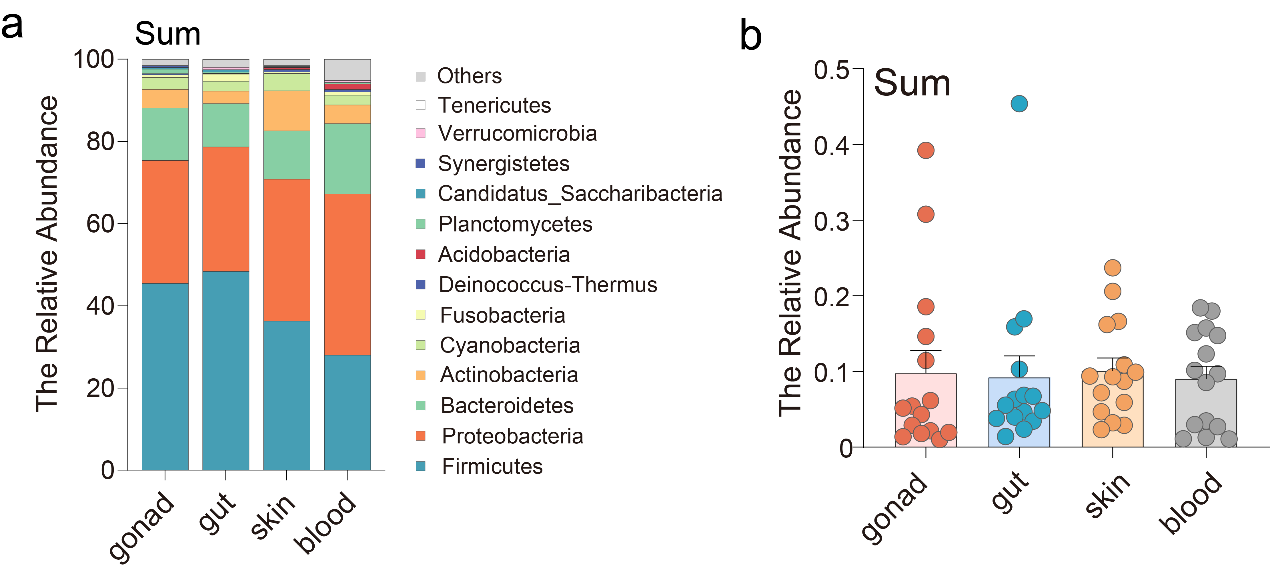


**Figure S2.** Microbiome signatures across different sites in swamp eel. **a** Stacking diagram showing the composition analysis of the bacterial community at the phylum level in swamp eel. **b** Representative abundance of *Bacillales* at the order level in swamp eel.


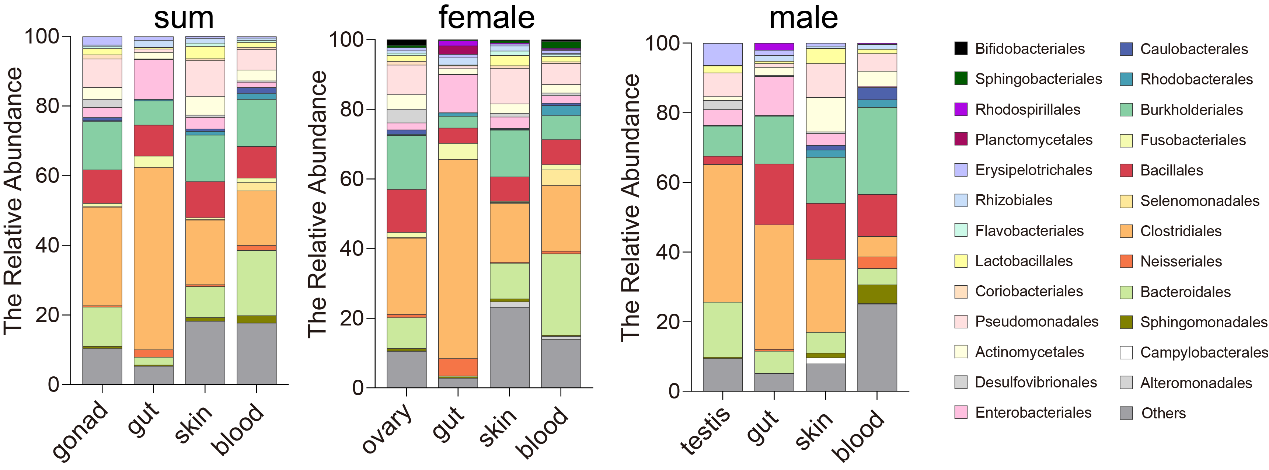


**Figure S3.** Stacking diagram showing the composition analysis of the bacterial community at the order level in all (left), female (middle), and male (right) swamp eel, respectively.


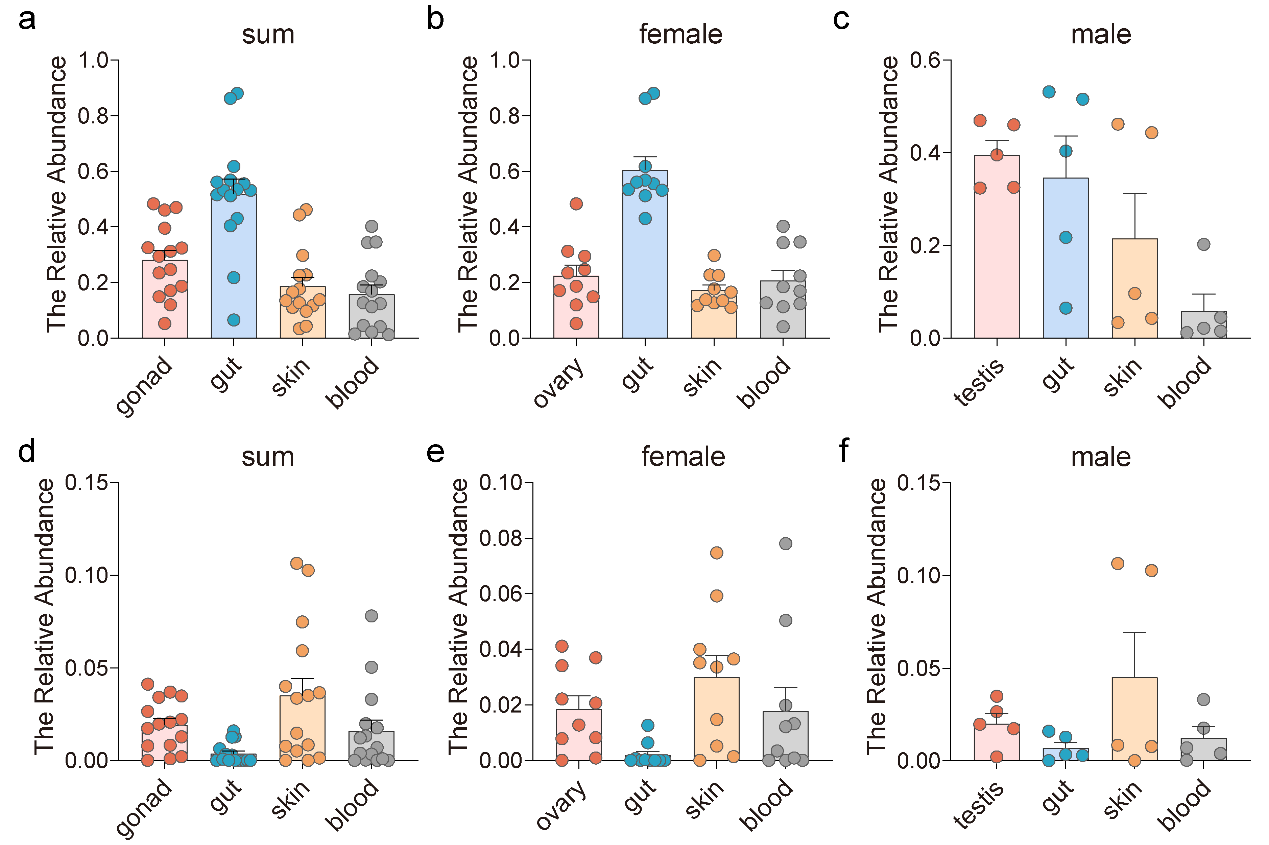


**Figure S4. a-c** Representative abundance of *Clostridiales* at the order level in all (a), female (b), and male (c) swamp eel, respectively. **d-f** Representative abundance of *Lactobacillales* at the order level in all (d), female (e), and male (f) swamp eel, respectively.


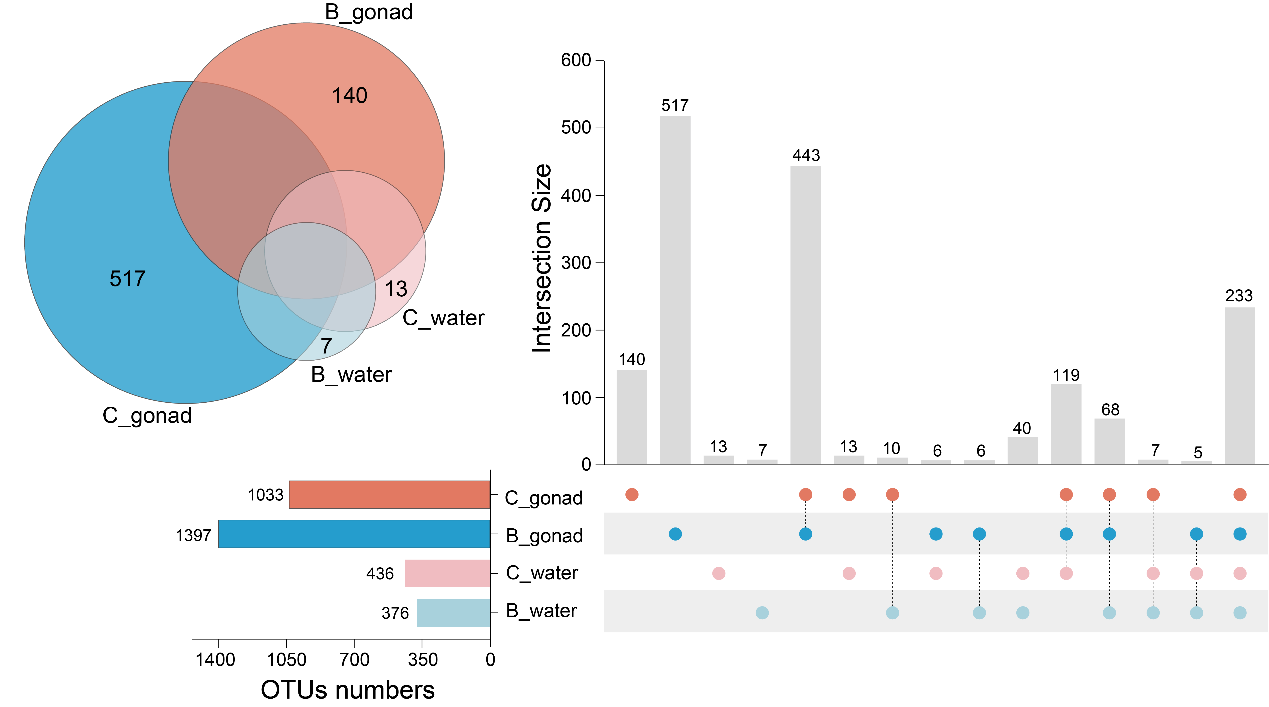


**Figure S5.** The Venn diagram and upset plot of microbiota in the water and ovaries after *Bacillus* injection.

**
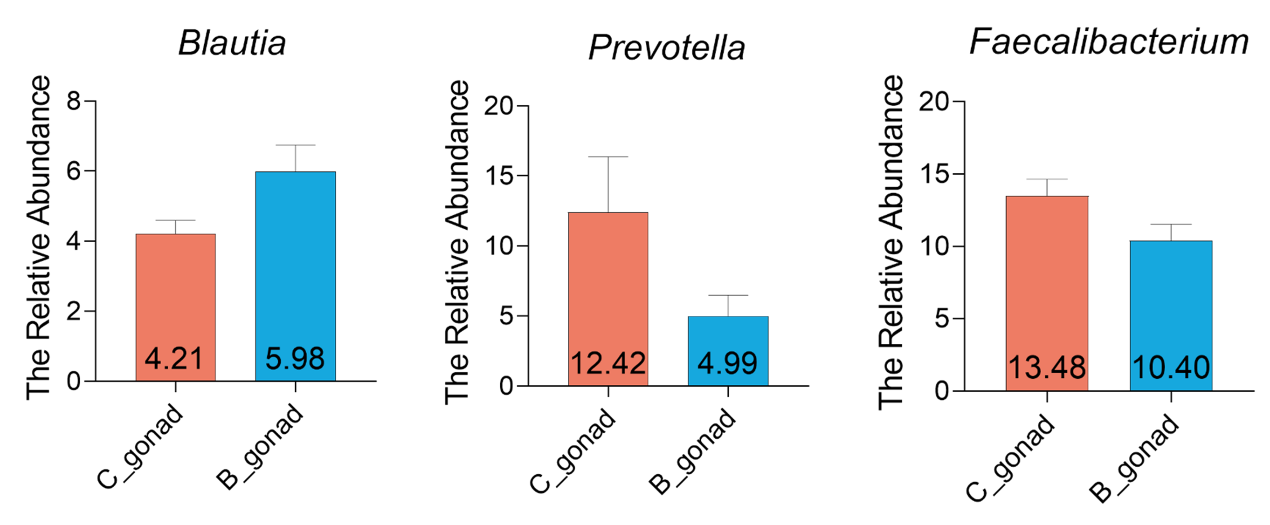
**

**Figure S6.** Representative abundance of *Blautia* (left), *Prevotella* (middle), and *Faecalibacterium* (right) at the genus level from control and *Bacillus*-injected ovaries.

**TABLE S1 |** The relative abundance of bacterial communities in different tissues of swamp eel.

|  | **Gonad** (%) | | | **Gut** (%) | | | **Skin** (%) | | | **Blood** (%) | | |
| --- | --- | --- | --- | --- | --- | --- | --- | --- | --- | --- | --- | --- |
|  | sum | female | male | sum | female | male | sum | female | male | sum | female | male |
| Tenericutes | 0.11 | 0.21 | 0.01 | 0.02 | 0.05 | 0 | 0.19 | 0.38 | 0 | 0.05 | 0.10 | 0 |
| Verrucomicrobia | 0.03 | 0.04 | 0.01 | 0.55 | 0.06 | 1.04 | 0.09 | 0.12 | 0.06 | 0.42 | 0.39 | 0.44 |
| Synergistetes | 0.37 | 0.75 | 0 | 0 | 0 | 0 | 0.03 | 0.06 | 0 | 0 | 0.01 | 0 |
| Candidatus_Saccharibacteria | 0.38 | 0.73 | 0.03 | 0.54 | 0.01 | 1.08 | 0.17 | 0.08 | 0.26 | 0.11 | 0.22 | 0 |
| Planctomycetes | 1.12 | 0.02 | 2.23 | 0.30 | 0.59 | 0 | 0.04 | 0.09 | 0 | 0.35 | 0.10 | 0.61 |
| Acidobacteria | 0.03 | 0.06 | 0.01 | 0.07 | 0.12 | 0.01 | 0.49 | 0.75 | 0.22 | 1.32 | 0.31 | 2.34 |
| Deinococcus-Thermus | 0.21 | 0.23 | 0.20 | 0.15 | 0.19 | 0.12 | 0.54 | 0.47 | 0.61 | 0.60 | 0.75 | 0.44 |
| Fusobacteria | 0.69 | 1.39 | 0 | 1.78 | 3.57 | 0 | 0.40 | 0.38 | 0.42 | 0.77 | 0.73 | 0.80 |
| Cyanobacteria | 2.87 | 3.80 | 1.94 | 2.34 | 2.28 | 2.40 | 4.21 | 6.90 | 1.51 | 2.42 | 3.87 | 0.96 |
| Actinobacteria | 4.52 | 6.12 | 2.93 | 3.07 | 3.00 | 3.13 | 9.73 | 9.73 | 9.74 | 4.50 | 4.12 | 4.89 |
| Bacteroidetes | 12.76 | 19.40 | 6.12 | 10.52 | 5.91 | 15.13 | 11.68 | 14.59 | 8.77 | 17.04 | 20.83 | 13.25 |
| Proteobacteria | 29.84 | 29.74 | 29.93 | 30.22 | 36.18 | 24.25 | 34.43 | 35.47 | 33.39 | 38.98 | 32.10 | 45.87 |
| Firmicutes | 45.43 | 35.87 | 54.99 | 48.30 | 45.17 | 51.42 | 36.25 | 28.84 | 43.67 | 27.91 | 32.58 | 23.23 |
| Others | 1.46 | 1.43 | 1.49 | 1.92 | 2.49 | 1.35 | 1.47 | 1.80 | 1.13 | 5.02 | 3.47 | 6.56 |

**TABLE S2 |** Primers used in this study.

| **Gene** | **Primer Sequence (5’-3’)** | | **Amplicon Length (bp)** | **GenBank accession no.** |  |
| --- | --- | --- | --- | --- | --- |
|  | **Forward primer** | **Reverse primer** |  |  |  |
| \| *bcl2* \| \| --- \| | AAATCACTACCGAACGAAC | CACCTCCGACTCTACCTG | 193 | XM_020608275.1 | |
| \| *caspase7* \| \| --- \| | TGGCACGGATATAGATGCAGG | GTCTTCCTTCGAGGCTTCTGTC | 127 | XM_020605231.1 | |
| \| *caspase9* \| \| --- \| | TGGCACTGAGGTAAGTCACAAC | GAAGGACATTTCTGCCCGCT | 111 | XM_020612566.1 | |
| \| *caspase3* \| \| --- \| | CCACAGTAGCACAGGGAT | CTCGGATACACTTGACAGAA | 151 | XM_020595425.1 | |
| \| *caspase2* \| \| --- \| | GGACTGTGGAGTGGAGCAAA | ATGGCTGCTGTGCAAATTCT | 207 | XM_020610781.1 | |
| \| *caspase6* \| \| --- \| | GGGACAAACCTGAAGGAAGTGC | CCTCGTCGTCTGTGGTTCATC | 151 | XM_020591841.1 | |
| \| *calpain1* \| \| --- \| | TGGGATCGTGATGAGGGTCT | ATCTCGGTCCAAGCTCGTTG | 166 | XM_020619725.1 | |
| \| *fadd* \| \| --- \| | GCCGACACAACGGAGTATCT | TTACCTCTGTGGCGATGTTC | 154 | XM_020613729.1 | |
| \| *bax* \| \| --- \| | CTTTGCCTGTCGGCTTGTCA | ATACCCTCCCAGCCACCTTG | 138 | XM_020607426.1 | |
| *gpx4* | CCAATGTTGCCTCCAAATGA | CGTTGCCTGGCTCCTGAT | 138 | XM_020612291.1 | |
| *gpx8* | GTCCACTTACGGTGTTACCT | ATGGGCTCGTCAGTTCTC | 180 | XM_020593975.1 | |
| *gpx1* | GTTCACCGCCAAACTCTT | TTCCCATTCACATCTACCTT | 303 | XM_020607739.1 | |
| *gstk1* | TTGATGTTCCCCTGCGTTAT | CACCTGCTCTACCTGCTTGTC | 131 | XM_020610780.1 | |
| *gsto1* | GGGAGAAATAAAGGTGAGGATG | CAGATGAGTTGACAAGGCAGTT | 199 | XM_020600427.1 | |
| *sod* | AGCTGGCTAAGTTCTCATTCAC | GCAGTAACATTGCCCAAGTCT | 227 | XM_020598413.1 | |
| *cat* | GTCCAAGTCTAAGGCATCTCC | CTCCTCTTCGTTCAGCACC | 106 | XM_020624985.1 | |
| *nrf2* | CTTCAGACAGCGGTGACAGG | GCCTCATTCAGTTGGTGCTT | 260 | XM_020596409.1 | |
| *keap1* | AGCCTGGGTGCGATACGA | CAAGAAATGACTTTGGTGGG | 198 | XM_020597068.1 | |
| *nf-kb* | ACCCTACCGTGACACTAACCT | TGCCGTCTATCTTGTGGAAT | 222 | XM_020616319.1 | |
| *il-6* | TCAACAAGCACTTCCCAGCA | TGGGCCAAGCATTGAGTACC | 213 | XM_020606850.1 | |
| *il-8* | TGAGTCTGAGAAGCCTGGGA | GCAGTGAGAGTTGGCAGGAA | 113 | XM_020597077.1 | |
| *il-1β* | GAGATGTGGAGCCCAAACTT | CTGCCTCTGACCTTCTGGACTT | 127 | XM_020585780.1 | |
| *gsdme* | TTGTATGGCGAAGCGTTGTT | ACTGGGATCAGAGAACCTCCA | 170 | XM_020612826.1 | |
| *caspase 1* | AACACGGCGTAGAGGATGAC | ACACTCCATGTTCCACACCC | 237 | XM_020589454.1 | |
| *nlrc3* | TTCACGACATCGACGCAAAT | CCATCCAGAAAGGAGGCTGT | 229 | XM_020606091.1 | |
| *il-12β* | CAAGTCAGTTGCCAAAATCC | CCAAGCAGCTCAGGGTCT | 103 | XM_020594580.1 | |
| *tnf-α* | TCTTCAACAGGCATGGCGAG | GCTGGGTTGTATTCACCTTCT | 121 | XM_020624826.1 | |
| *rpl-17* | AACGCTGAACTGAAGGGTCTC | CATGTAGGGATTGATGCGGC | 120 | XM_020587712.1 | |
| *ef-1α* | CGCTGCTGTTTCCTTCGTCC | TTGCGTTCAATCTTCCATCCC | 102 | XM_020588923.1 | |

**TABLE S3 |** The sperm parameters related to total motility of swamp eel.

|  | **Curvilinear velocity**  **(VCL, μm/s)** | | **Average path velocity**  **(VAP, μm/s)** | | **Straight line velocity**  **(VSL, μm/s)** | |
| --- | --- | --- | --- | --- | --- | --- |
|  | Sperm+Medium | Sperm+*Bacillus* | Sperm+Medium | Sperm+*Bacillus* | Sperm+Medium | Sperm+*Bacillus* |
| 1 | 65.86 | 17.19 | 28.62 | 14.59 | 21.83 | 13.77 |
| 2 | 72.02 | 40.21 | 40.32 | 22.42 | 30.90 | 16.98 |
| 3 | 71.81 | 27.19 | 37.55 | 16.76 | 25.52 | 9.26 |
| 4 | 111.16 | 42.00 | 51.99 | 20.88 | 31.86 | 11.64 |
| 5 | 116.59 | 37.76 | 53.38 | 21.62 | 28.23 | 16.16 |
| 6 | 58.39 | 32.6 | 31.46 | 18.04 | 25.26 | 12.33 |
| 7 | 62.57 | 53.96 | 36.73 | 27.86 | 21.67 | 20.45 |
| 8 | 68.53 | 22.16 | 32.95 | 17.10 | 21.51 | 15.54 |
| 9 | 42.44 | 37.52 | 20.64 | 22.64 | 15.70 | 14.96 |
| 10 | 43.01 | 16.42 | 20.81 | 11.44 | 16.09 | 9.54 |
| 11 | 41.09 | 16.17 | 24.49 | 11.72 | 18.78 | 9.91 |
| 12 | 79.34 | 22.67 | 36.95 | 16.49 | 25.24 | 15.48 |
| 13 | 25.55 | 35.45 | 18.94 | 21.15 | 15.66 | 17.19 |
| 14 | 73.73 | 12.76 | 35.5 | 9.73 | 26.95 | 8.80 |
| 15 | 50.69 | 12.25 | 31.51 | 8.37 | 27.36 | 7.51 |
| 16 | 68.33 | 7.53 | 25.36 | 5.37 | 10.64 | 4.82 |
| 17 | 21.98 | 18.85 | 14.11 | 13.71 | 9.76 | 11.03 |
| AVE+S.E.M. | 63.12 ± 24.69 | 26.63 ± 12.57 | 31.84 ± 10.50 | 16.46 ± 5.78 | 21.94 ± 6.45 | 12.67 ± 3.99 |
